# Diagnosis of hepatic glycogen storage disease patients with overlapping clinical symptoms by massively parallel sequencing: a systematic review of literature

**DOI:** 10.1186/s13023-020-01573-8

**Published:** 2020-10-14

**Authors:** Zahra Beyzaei, Bita Geramizadeh, Sara Karimzadeh

**Affiliations:** 1 Transplant Research Center, Shiraz University of Medical Sciences, Shiraz, Iran; 2 Department of Pathology, Shiraz University of Medical Sciences, Zand St., Shiraz, Iran; 3 Shiraz Medical School Library, Shiraz University of Medical Sciences, Shiraz, Iran

**Keywords:** Glycogen storage disease (GSD), Massively parallel sequencing, Exome sequencing, Targeted gene sequencing, Rare disease diagnosis, Genetic diagnosis

## Abstract

**Background:**

Glycogen storage diseases (GSDs) with liver involvement are complex disorders with similar manifestations. Currently, the main diagnostic methods such as tissue diagnosis, either histopathology or enzyme assay, are invasive. Meanwhile, GSDs are diseases with significant genetic heterogeneity, and gene-sequencing methods can be more useful. This systematic review aims to review the literature to assess the value of massively parallel sequencing in the diagnosis of GSDs on patients with previously undiagnosed hepatic involvement.

**Methods:**

Relevant studies identified in the MEDLINE/PubMed, EMBASE, Cochrane Library, Scopus, and Web of Science Core Collection databases up to July 2019 with no time and language restrictions. Publications were included in the review if they analyzed GSDs with hepatic involvement (GSD I, GSD III, GSD IV, GSD VI, GSD IX), using targeted gene sequencing (TGS) or exome sequencing (ES).

**Results:**

Eleven studies were included in this systematic review. ES demonstrated a 93% diagnostic yield. These methods correctly distinguished all types of pathogenic variants. The diagnostic yield of the TGS method was around 79.7%.

**Conclusions:**

According to our results, TGS analysis can be considered as the first-line diagnostic method with valuable results and ES can be used to diagnose complex cases of GSD with liver involvement. Overall, these molecular methods are considered as accurate diagnostic tools, which expedite correct diagnosis and treatment with significant cost-effectiveness by reducing unnecessary and inaccurate tests.

**PROSPERO registration:**

CRD42020139931. Registered 8 January 2020.

## Background

Glycogen storage disorders (GSDs) are a group of rare metabolic diseases with abnormal glycogen metabolism. The incidence of GSD is approximately 1:10,000 live births. These groups of diseases are caused by various enzyme deficiencies resulting in abnormal glycogen synthesis, or glycolysis, typically within the muscles and/or liver cells [1, 2]. Different types of GSDs are categorized based on the type of deficient enzymes and affected tissues [3].

GSDs with liver involvement (Hepatic GSDs) are a complex group of disorders, including GSD Ia (*G6PC*, MIM # 232200), Ib (*SLC37A4*, MIM # 232220), III (*AGL*, MIM # 232400), IV (*GBE1*, MIM # 232500), VI (*PYGL*, MIM # 232700), IXa (*PHKA2*, MIM # 306000), IXb (*PHKB*, MIM # 261750), and IXc (*PHKG2*, MIM # 613027). All of them are associated with hypoglycemia and hepatomegaly [2]. Clinical signs of different types of hepatic GSDs are very similar, such as short fasting intervals (less than 4 h), hepatomegaly or hypoglycemia, which can be observed in GSD type I as well as GSD III, IV, VI and IX. However, treatment methods and modalities, complications, and natural histories are different in various types of GSDs, which prompts definite and differential diagnosis between various types of GSDs. This can help to improve the quality of life, by decreasing the end-organ damage [3, 4]. Moreover, these diseases create significant expenses for healthcare systems, which also prompts the necessity of precise diagnostic methods [5].

Currently, for accurate diagnosis of hepatic GSDs, a liver biopsy must be performed. Although the role of liver biopsy in the diagnosis of GSD is less common, however many publications still consider liver biopsy as the gold standard. Enzyme assay in the liver tissue is another option that can be measured for definite and decision-making diagnosis. Although the enzymatic activity of glucose-6-phosphatase (*G6PC*) for GSD Ia can be performed on frozen liver tissue, measuring glucose-6-phosphate translocase (*G6PT1*) activity for GSD Ib is difficult to be performed on frozen liver samples and needs fresh liver tissue. Both of these necessitate liver biopsy, which is an invasive procedure, so the majority of clinical diagnostic laboratories do not assay the latter enzyme activity [2]. In addition, measurement of G6PC and G6PT1 as well as glycogen phosphorylase (*PYGL*) activity in the tissue samples is very laborious and requires a sophisticated laboratory. Molecular tests have rarely been used as the first diagnostic method or the method of choice in previous study reports [4, 6].

All hepatic GSDs, except for GSD IXa, are autosomal recessive. GSD IXa is an X-linked recessive disorder, so, molecular methods may provide a suitable procedure for the diagnosis and classification of hepatic GSDs. For many years, the conventional Sanger sequencing method has been the gold standard for the detection and screening of mutations. However, this method can only evaluate the exon-by-exon of one gene at a time, and some exons require multiple Sanger steps. Therefore, its validation for a mutational screening of large genes such as *AGL* with 34 exons; the corresponding controls and necessary bidirectional reads would be very laborious [7]. In addition, due to the genetic heterogeneity of populations, consecutive tests of every candidate gene are costly and time-consuming, leading to a delayed diagnosis that decelerates care and treatment [2].

Compared with other molecular methods, massively parallel sequencing (MPS), also known as next-generation sequencing has the ability to simultaneously screen of large numbers of genes. It also adds unique gene sequence tags to each sample, allowing pooled testing and preventing invasive liver biopsies [2–8]. This pooling lets different patients to be sequenced together with simultaneous detection of other genomic alterations, e.g. screening GSD-associated genes and similar non-GSD-associated genes in one panel [7, 8]. Since 2009, MPS has been used for exome sequencing (ES), allowing targeted gene panels (TGS) to be sequenced faster in higher depth, which increases the sensitivity [9–11]. Despite all recent advances in MPS, the cost of sequencing is still remarkable and is different for various types of MPS technology [12]. Moreover, over time, there has been a significant decrease in sequencing costs, which has made the clinical application of MPS more practicable [13–15]. Another important issue that must be considered is the depth of sequence coverage [16]. It is a fact that higher coverage of sequencing increases the validation of findings as well as costs. Therefore, investigators try to design clinical experiments with the best accuracy, coverage, and cost.

The whole evidence in the MPS application in patients with hepatic GSD has not been previously reviewed systematically. Therefore, it can be worthwhile to provide the best and most reliable objective analysis of the existing evidence from previous reports. For this purpose, we systematically reviewed the existing literature to assess the value of MPS in the diagnosis of GSDs in patients with previously undiagnosed hepatic involvement [2, 7, 17–25]. The review focused specifically on GSDs with hepatic involvement (GSD I, GSD III, GSD IV, GSD VI, GSD IX) diagnosed with exome sequencing (ES) or targeted gene sequencing (TGS).

## Materials and methods

We conducted this study, according to the Preferred Reporting Items for Systematic Reviews and Meta-Analyses (PRISMA) guidelines [26] (Additional file 1). A complete protocol was registered at PROSPERO under the number CRD42020139931.

MPS is described to include ES and TGS. The ES panel consisted of all known associated disease genes available in the OMIM database until 2013. The TGS panel included all known genes in metabolic disorders, including GSD-associated genes, or only GSD-associated gene-disease panels with/or without genes related to its pathologic phenotypes panels. Different studies reported MPS only for probands or probands alongside their parents, siblings, or grandparents (duo or trio). This study is mainly based on the utilization of MPS for the diagnosis of GSDs with hepatic involvement.

### Search strategies and data sources

We conducted an independent review of MEDLINE/PubMed, EMBASE, Cochrane Library, Scopus, and Web of Science Core Collection databases with no time and language restrictions on November 30, 2018 and updated on July 31, 2019. The bibliography of the selected articles on the topic was manually searched for additional studies and for minimizing publication bias. The search strategy was designed and implemented by an experienced medical librarian using controlled keywords and the MeSH terms (Medical Subject Heading) from the Library of Shiraz Medical Center. One word (keyword) was identified by examining relevant references in the literature and the Medical Subject Headings (MeSH) used by EMBASE and MEDLINE (https://www.nlm.nih.gov/mesh/). The details of the search strategy are reported in Additional file 1.

### Inclusion and exclusion criteria

Studies were included if they met the following criteria:

1. Peer-reviewed original research articles related to hepatic glycogen storage disease, including type I, III, IV, VI, IX, which has been diagnosed by MPS.
2. Case series related to hepatic glycogen storage disease, including type I, III, IV, VI, IX, which has been diagnosed by MPS.
3. Description/evaluation of the clinical application of MPS for diagnostic purposes with a proband. We also included articles related to carrier testing for hepatic GSDs, prenatal genetic testing, and targeted gene sequencing (e.g., “clinical exome” or “Mendeliome”), i.e. panels of thousands of genes known to be associated with single-gene disorders, provided they have included genes related to hepatic GSDs. There were no restrictions in selecting papers relative to their study design, including interventional studies (any methodology), and clinical reports (case series). Studies were excluded if they met the following criteria:
  1. All studies reporting the use of MPS in the diagnosis of other types of GSDs, such as muscular forms (type III, IV) (i.e. not including hepatic GSDs).
  2. All publications which have used only mitochondrial genome sequencing (i.e. without sequencing of the nuclear genome).
  3. All the animal experiments, editorial pieces, commentaries, review articles, and symposium reports.
  4. All case reports because these studies cannot determine the sensitivity and specificity of the tests.

Conference abstracts were included and evaluated in the protocol although none of them was eventually eligible. The literature search was undertaken in November 2018 and all citations were imported into EndNote (Clarivate Analytics, Boston, MA). Following deduplication, publications were scanned for relevance by title and abstract. Clearly, irrelevant publications were excluded. The full text of the remaining publications was then evaluated for relevance by both authors (ZB and BG) independently. The selected studies were comprehensively surveyed by both reviewers, and those, which fulfilled the eligibility criteria were selected for detailed data mining and the quality assessment. Disagreements at both stages were resolved by consensus and referring back to the original article.

### Methodological quality assessment

Both authors (ZB and BG) independently conducted a quality assessment of the studies. For quality assessment, checklists were used which have been developed by the National Heart, Lung, and Blood Institute (NHLBI) Quality Assessment Tool for Observational Cohort and Cross-Sectional Studies. Cochrane (NHLBI 2014) has recommended these checklists [27]. The tools assessed each quality criterion as “Yes,” “No,” or “cannot be determined” “Not reported” or “Not applicable”. Reviewers categorized them as: > 8 yes = Good, 7–8 yes- Fair, and < 6 yes = Poor. The total agreement between the reviewers was 82% as shown in Additional file 1. Meanwhile, as all the included articles were observational studies, the context and population structure were also considered.

### Data extraction

According to the PRISMA guidelines, data extraction was independently carried out by the two authors (ZB, BG), using a data extraction form. Disagreements were resolved by consensus; if not, the original article was evaluated once more, and finally, the issue was resolved through discussion to reach the consensus. The collected data included bibliographic details, information about the first author name/year of publication, type of study/GSD, number of patients, presentation of disease, country, mean age of the patients at molecular diagnostic test, consanguinity, measurement of enzyme activity, liver biopsy type of MPS/panel, sequencing methods, sequencing platforms, control database, MPS instrument brand, whether a duo and/or trio approach was used. The authors contacted the corresponding author of selected articles to get access to more details, if needed. The analysis was performed with Stata IC 15 (College Station, TX).

## Results

### Study selection

**Fig. 1:**
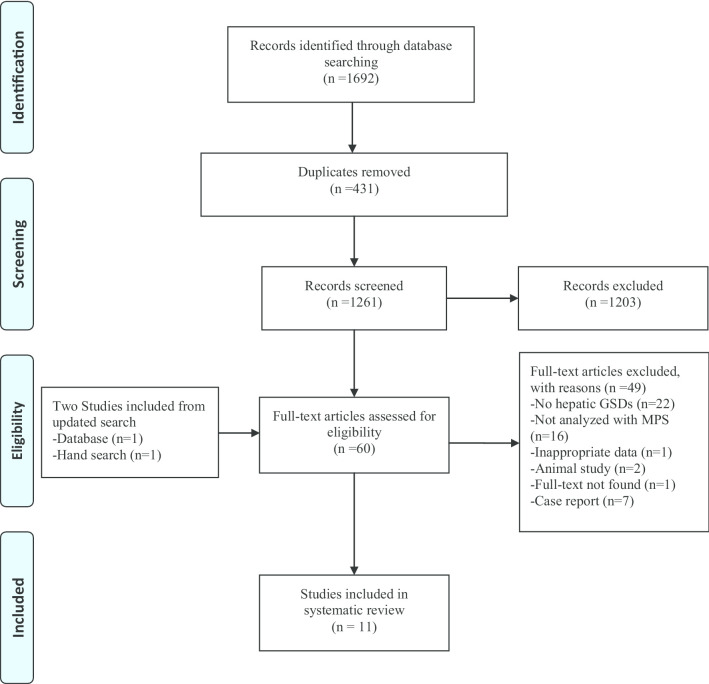
The flow diagram of the study selection for the systematic review

The study selection process is presented in Fig. 1 as a PRISMA flow diagram. In primary search, 1692 articles were identified, of which 431 articles were duplicated. After initial screening of the titles and abstracts, 1203 articles were excluded based on the selection criteria and 58 full text articles as well as two studies from updated search were assessed for eligibility (n = 60). In addition, the inter-rater reliability was measured by Cohen’s kappa coefficient (*K* = 0.85 ± 0.02) which has shown good agreement with the inclusion/exclusion criteria between the reviewers [28]. Following the review of the remaining articles and resolution of discrepancies by consensus among reviewers (ZB and BG), 11 studies were finally included in this systematic review.

### General characteristics of the study

**Fig. 2:**
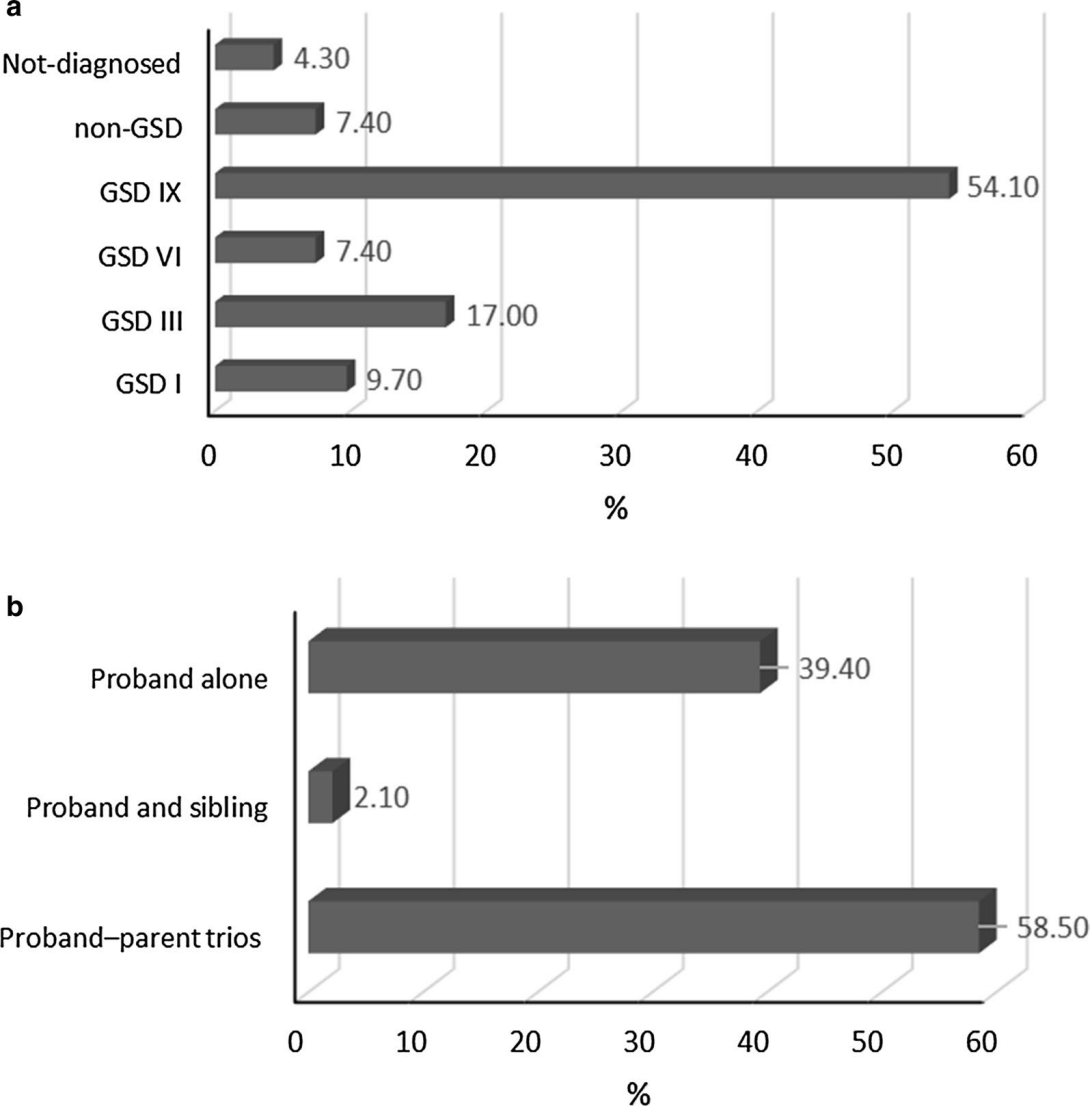
The percentage of patients was diagnosed with MPS method (**a**) and the percentage of Trio-based test was performed (**b**)

We analyzed 11 studies [2, 7, 17–25] with 94 hepatic GSD patients (72 Male, 22 Female). The detailed characteristics and outcomes of the included studies are summarized in Table 1. All eleven studies analyzed in this article were published between 2013 and 2019, and contained 5 retrospective cohorts [7, 17, 20, 23, 24], 4 cross-sectional [2, 18, 19, 21], and 2 case series [22, 25]. Five studies [17–19, 24, 25] included Asian participants, such as Chinese, Korean and Qatari, and the remaining 6 studies [2, 7, 20–23] included Western participants, including 2 American, 1 Serbian, 1 Spanish, and 2 Canadian. Those 94 participants analyzed by MPS included hepatic GSD disease patients (2 GSD Ia, 7 GSD Ib, 16 GSD III, 7 GSD VI, 42 GSD IXa, 4 GSD IXb, 5 GSD IXc) and 7 non-GSD patients, and 4 patients with no exact diagnosis (Fig. 2a). GSD type IV participants with neuromuscular involvement and without liver involvement were excluded. The mean age of the disease onset was 2.1 years (Ranges: 1–35 months), and at the time of the molecular diagnostic test it was 5.8 years (Ranges: 10 months to 41 years).

**Table 1:** Main characteristics and outcomes of included studies

First author/year [Ref.]	Study design	No. (M:F)	Presentation	Mean age at mol. Diag (Y)	Country/ethnicity	SS (< 3%)	Parent marriage	Enzyme activity	Liver biopsy	MPS type/panel	Suspected disease	Result of molecular test	Read length	Mean depth of coverage	Type of sequencer	Trio-based test (parents)
Tong et al. [17]	Coh	2 (1:1)	Developmental delay, Hepatosplenomegaly, Dystrophia, Neutropenia,	2.5	China	2 patients	Non-consanguineous	NR	NR	ES/ Exome Sequencing TruSight One Gene Panel	Neurodevelopmental Disease	1 GSD Ia, 1 GSD Ib	2 × 100–150 bp	142×	HiSeq2500 (Illumina)	YES Both parent
Roscher et al. [20]	Coh	21 (17:4)	Hepatomegaly, liver fibrosis and adenoma, mild cardiomyopathy	11.7	Canada	2 patients	Non-consanguineous	YES: 14 patients	YES: 10 patients	TGS/NA	GSD III or GSD VI	11 patients GSD IXa; 3 patients GSD IXb; 3 patients GSD IXc; 4 patients GSD VI	2 × 100 bp	100×	HiSeq2000 (Illumina)	YES both parents
Skakik et al. [23]	Coh	5 M	Hepatomegaly and hypoglycemia	1.3	Serbia	YES	Non-consanguineous	YES	YES	ES/ Exome Sequencing TruSight One Gene Panel	Hepatic GSD	GSD III, VI, IXa as well as in non-GSD associated genes, *LIPA* and *SBDS*, responsible for cholesteryl-ester storage disease and Schwachman-Diamond syndrome respectively	2 × 100 bp	100 ×	MiSeq (Illumina)	NO
Vega et al. [7]	Coh	22(13:9)	Hepatomegaly, dysmorphic facies, hypoglycemia, hyperuricemia, hyperlipidemia and kidney failure, hypertransaminasemia,	13.5	Spain	YES	NR	NR	NR	TGS/Metabolic disorders panel (Agilent) ES/ Exome Sequencing TruSight One Gene Panel	GSDs	11 NOT detected by TGS:4 Detected (GSD III, VI, IXb, *ALDOB*) 18 repeated and detected by ES (1 GSD Ib, 6 GSD III, 7 GSD IXa) as well as in not GSD associated genes, *LIPA, CPT2, ANO5, NKX2-5*	2 × 250 bp 2 × 250 bp	400× 83.6×	MiSeq (Illumina)	YES parents
Zhang et al. [24]	Coh	17 M	hepatomegaly, growth retardation, and liver dysfunction	9.9	China	7 YES, 10 NO	Non-consanguineous	NO	8 YES, 9 NO	TGS/ GSD panel (Agilent)	GSD IX	17 GSD IXa	2 × 100 bp	100×	HiSeq2000 (Illumina)	NO
Wang et al. [2]	CS	16(9:7)	Hypoglycemia and mild hepatosplenomegaly, lactic acidosis, neutropenia	6.5	USA	YES	15 Non-consanguineous:1 Consanguineous	YES	YES	TGS/ GSD panel (Agilent)	GSDs	8 detected and matched with signs (1 GSD Ia, 2 GSD Ib, 3 GSD III, 2 GSD IXa), 4 detected truly with TES but direct seq. not found mut (1 suspected as GSD Ia, recognized as GSD Ib; 1 suspected as GSD 0 recognized as GSD IXc.; 1 suspected as GSD III recognized as GSD VI; 1 suspected as GSD VI or IX recognized as GSD IXa, 5 suspected as hepatic GSD but NOT detected by TGS	1 × 100 bp	758×	HiSeq2000 (Illumina)	5 YES Both parents, 11 NO
Wang et al. [21]	CS	3 M	Hepatomegaly and hypertriglyceridemia	7.3	USA	NO	Non-consanguineous	NR	NR	ES/NA	Hepatic GSD	3 GSD III	1 × 100 bp	~ 1000×	HiSeq2000 (Illumina)	NO
Choi et al. [19]	CS	2 M	Hepatomegaly, elevated AST and ALT levels, neutropenia	0.83	Korean	YES	Non-consanguineous	YES	YES	ES/NA	GSD I	2 GSD Ib	2 × 150–200 bp	NR	HiSeq2000 (Illumina)	NO
Fahiminiya et al. [18]	CS	1 M	Hepatomegaly, and recurrent hypoglycemia	6	Qatar	YES	Consanguineous	YES	YES	ES/NA	GSD I or III	1 GSD IXc	2 × 100 bp	100×	HiSeq2000 (Illumina)	NO
Rousseau-Nepton et al. [22]	C- series	2 (1:1)	Abdominal distension with hepatomegaly, difficulty walking	1.4	Canada	YES	Non-consanguineous	YES	NO	ES/NA	GSD I or III	2 GSD III	NR	100x	HiSeq2000 (Illumina)	YES Parents and siblings
Yang et al. [25]	C-series	3 M	Increased transaminase, Hepatomegaly, Hypoglycemic	2.8	China	1YES, 2 NO	1Consanguineous, 2 Non-consanguineous	YES	1 YES	TGS/ 300 genes associated with hepatopathy panel	Metabolic liver disorders	3 GSD IXa	NR	100×	HiSeq2500 (Illumina)	YES Parents and siblings

*Coh* cohort, *CS* cross-sectional, *C-Series* case-series, *NR* not reported, *SS* short stature, *MUT* mutation, *ES* exome sequencing, *TGS* targeted gene sequencing, *NA* not associated

The families of 69 patients, i.e. 96% (69/72) were non-consanguineous. Enzyme activity was analyzed in 46% (43/94) of patients. There was no report of enzyme assay in 28.7% (27/94) of the patients. All results described above are presented in Table 1.

### Synthesis of results

ES was used in 54.5% (6/11) of articles [17–23], TGS in 36.4% (4/11) [2, 20, 24, 25], and a combination of TGS and ES in 9.1% (1/11), as shown in Table 1 [7]. Seven studies [2, 18–22, 24] used Illumina Hiseq 2000; one study [17] used Illumina Hiseq 2500 and in two studies [7, 23] Miseq Illumina was the sequencing platform. Two out of 11 studies analyzed single-end reads with an average depth of > 600× [2, 21], while all other studies analyzed paired-end reads with an average depth of > 100×. As presented in Table 1, sequence analysis of proband–parent trios was performed in the majority of patients 58.5% (55/94); as also a duo of proband and sibling was reported in 2.1% (2/94) of patients [7, 17, 20, 22, 25]. 39.4% (37/94) of investigations were reported analyzing the proband alone (Fig. 2b).

Eighty-four percent (79/94) of patients were analyzed with TGS and sixteen percent (15/94) using ES methods. Our results showed that the overall diagnostic rate of ES, and TGS was 93% (14/15), 79.7% (63/79) for the detection of mutations in hepatic GSDs patients, respectively. Our findings demonstrated that by using ES methods, 100% of five patients with complex features were identified with a mutation in a GSD disease-associated gene although those patients were initially diagnosed as suffering from neurodevelopmental or other metabolic disorders. By application of the ES method, common mutations in the 5 patients were diagnosed with the non-GSD-associated disease, which was incorrectly diagnosed as hepatic GSDs [7, 23]. The detected genes were *LIPA* and *SBDS*, *CPT II*, *ANO5,* and *NKX2-5* which are the genes responsible for cholesteryl-ester storage disease, Schwachman-Diamond syndrome, carnitine palmitoyl transferase II deficiency, muscle disease (Limb-girdle muscular dystrophy type 2L and Miyoshi muscular dystrophy 3) and congenital heart disease, respectively.

Three studies [20, 24, 25], analyzing hepatic GSD patients with the TGS method, found that among 41 patients, 30 could be detected with mutations in the average depth of sequence ≥ 400× and average diagnostic yield of approximately 73.1% (30/41). It is noted that by increasing the sequencing average depth to > 1000×, the diagnostic yield of TGS could be enhanced to 86.8% (33/38). Also, the diagnostic yield of TGS by performing a trio-proband test could be increased from 64.5 to 79.7%. In 20.3% of patients (16/79) analyzed by the TGS method, no mutations were diagnosed.

Finally, forty-six percent of patients (43/94) had undergone liver biopsy before the molecular genetic test which was performed in forty-four percent (35/79) of TGS patients and fifty-three (8/15) of ES patients. There was no liver biopsy for 25.4% (24/94) of the patients. In 28.7% (27/94) of the patients, nothing was reported about the performance of liver biopsy. However, the features of liver histopathology in 41.8% (18/43) of patients were not consistent with the molecular genetic investigations. In 10 patients, features of liver histopathology were suggestive of GSD-III, but molecular genetic investigations confirmed the diagnosis of GSD-IXa in 4, diagnosis of GSD-IXc in 2, and diagnosis of GSD-VI in 4 patients. In 2 patients, features of liver histopathology were suggestive of GSD-IV, but molecular genetic results confirmed the diagnosis of GSD-IXa in 1 patient and diagnosis of GSD-VI in another patient. In addition, features of liver histopathology were suggestive of GSD-Ia in one, GSD-0 in one patient, and GSD-VI in one patient, but molecular genetic investigations confirmed the diagnosis of GSD-Ib in one, GSD-IXc in one, and GSD-IXa in one patient, respectively. In 3 patients, features of liver histopathology were suggestive of liver disorder and hepatic GSD without exact sub-typing, but molecular genetic investigations confirmed the diagnosis of GSD-IXa in one, cholesteryl-ester storage disease in one, and Schwachman-Diamond syndrome in one patient, respectively.

## Discussion

In this systematic review of 11 studies, our goal was to determine the diagnostic value of MPS as the first method of choice in GSDs with liver involvement. According to the results, most patients with hepatic GSDs are not provided with a specific molecular diagnostic test as the first approach of choice. The increased mean age of patients in disease onset in comparison with the time of a molecular performance from 2.1 to 5.8 emphasizes that this method has not been used as the first-line diagnostic method.

The standard approach for the diagnosis of hepatic GSDs is to identify specific phenotypic and clinical presentation of the disease, with consideration of liver biopsy findings, as well as of the tissue enzyme assay [1, 2, 29]. The main features of hepatic GSD are hepatomegaly and hypoglycemia, which delay the diagnosis and lead to chronic liver disease, e.g. fibrosis and cirrhosis [29]. It is worth noting that delayed diagnosis could have a harmful outcome in patients and their families leading to delayed treatment, and delayed recognition of the risk of recurrence in later pregnancies to protect younger siblings of the proband. In addition, other diagnostic methods can be time-consuming, invasive, and costly. Based on our results, the feature of liver histopathology may be a powerful and effective method for monitoring long-term liver complications, but not for confirming the diagnosis and accurate sub-typing. Therefore, the development of molecular method based on MPS may be valuable for an accurate diagnosis [30, 31]. It should be noted that the clinical availability of MPS methods is limited and is only implemented in developed countries [32]. This is because of the high cost of the procedure.

According to our results, 54.5% of the studies opted to look for mutations by exome sequencing (ES), which is considered as an effective method with 100% sensitivity for determining unknown coding mutations. All studies surveyed were carried out with the same mean coverage of the ES method (100-150x), which suggests an appropriate mean coverage for ES. Furthermore, with the discovery of the causative gene, ES is also an effective diagnostic tool whenever no diagnosis could be made or an incorrect diagnosis has been reported based on clinical manifestations [33]. For example, our results demonstrate that ES could identify 73.3% of the mutations in the disease-associated gene although the patient was suspected to have other types of GSDs based on the preliminary clinical diagnosis. Diagnosing the correct type of hepatic GSDs not only influences the prognosis and care but also allows suitable genetic counseling to the family [34]. Our results demonstrate the role of ES in the detection of novel variants of complex features of hepatic GSDs, and advocate a role for trio-ES in detection of unknown variants. On the other hand, the use of ES panels increases the rate of accurate diagnosis [35, 36]. The ES method turned out to be the test with the highest diagnostic yield, especially when accompanied by a trio-based test, with 93% sensitivity, as reported earlier [21]. Likewise, performing ES methods using an Illumina Clinical-Exome Sequencing TruSight One Gene Panel identified the patients with non-GSD disease. According to studies reported these genes (*LIPA*, *SBDS*, *CPT II*, *ANO5*, *NKX2*) which had mutations, screened by ES methods. Those genes are responsible for cholesteryl-ester storage disease, Schwachman-Diamond syndrome, carnitine palmitoyl transferase II deficiency, muscle disease (Limb-girdle muscular dystrophy type 2L and Miyoshi muscular dystrophy 3), and congenital heart disease respectively. These genes have no demonstrated role in GSDs, and they are manifested with overlapping phenotypic characteristics, such as hypoglycemia, seizures, hepatomegaly, cardiomyopathy, and arrhythmia.

The other method of MPS, TGS also detected pathogenic variants but its sensitivity was very different. It is noted that TGS and ES are used to perform targeted exome sequencing of metabolic disorders, including GSDs-associated genes with/or without genes related to its pathological phenotypes and all known disease-associated genes, rather than the entire exome, respectively [37–39]. Our results show that the diagnostic rate of TGS is 79.7%, though the rate may be affected by the type of panel selected, depth of sequencing coverage, and whether other family members are analyzed. The selection of the appropriate panels covering a wide range of similar GSD-genes is very important which shows different diagnostic yields for the TGS method in different reports [2, 7, 20, 24, 25]. It is probably explained by the high resemblance of GSD to other non-GSDs disorders with phenotype overlapping. In the undiagnosed cases by TGS, the mutations probably existed in coding regions that were not adequately covered, or they could be located within deep intronic regions that were not covered by TGS or might be within non-GSD-associated genes. Also, we found that increasing the depth of the sequence coverage enhanced the diagnostic yield with the TGS method. Previous investigations have shown that diagnostic yield increases by performing capture-based enrichment, followed by deep sequencing (1000x) [2]. Capture/MPS allows detection of a wide spectrum of mutations [21]. This technology helps to detect all types of mutations such as single nucleotide substitutions, small insertion/deletions as well as exonic copy number variation, and large genomic rearrangements [38]. Our results also showed that analysis of trios performed in 60.6% of the patients, significantly enhanced the diagnostic yields, compared with proband-only testing, due to the heterogeneous genetic basis of hepatic GSDs. Consequently, the TGS method might be suitable for first-line molecular study of hepatic GSDs, and it is recommended to be performed only when presentations of disease are very clear, using a wide range of disease panels.

**Fig. 3:**
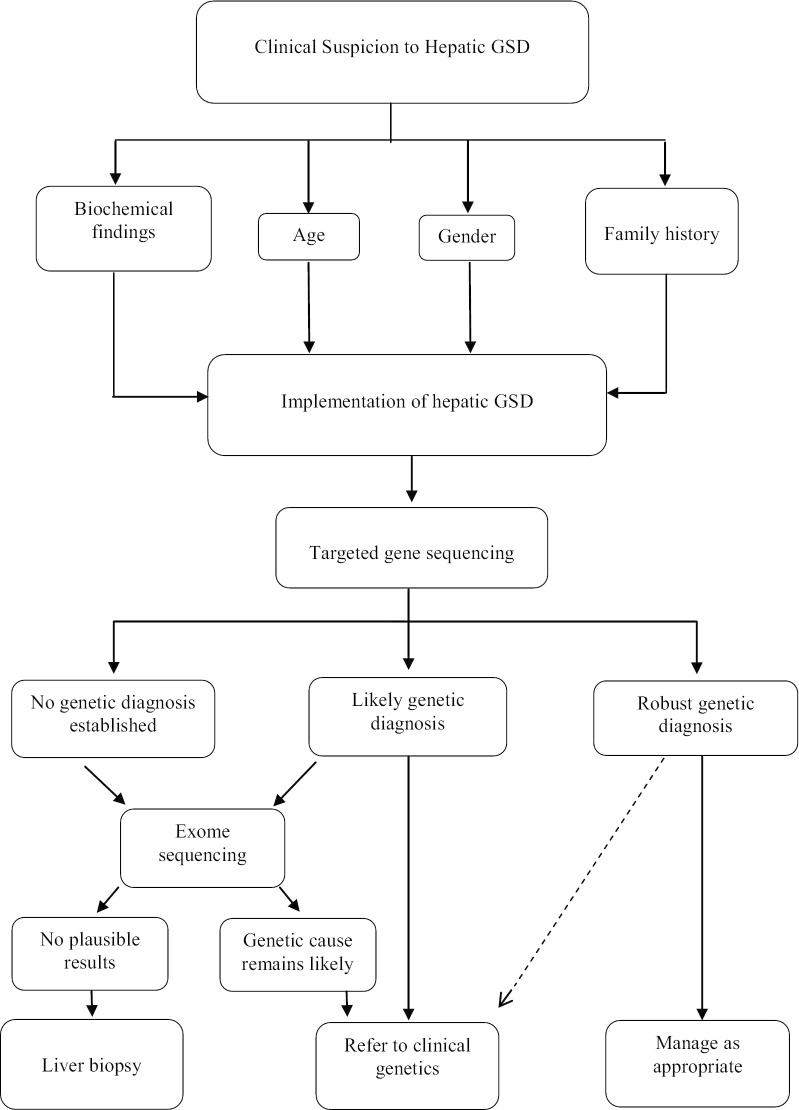
Integration of clinical and laboratory workflows to optimize the hepatic glycogen storage disease diagnosis

Therefore, the best diagnostic strategy to identify hepatic GSDs can be starting with a TGS method, as a more cost-effective method than the ES, but with the high coverage and a wide range of the panel. If there is no definite result, then analysis with a more comprehensive method, such as an ES, should be performed [40]. ES should particularly be the diagnostic tool of choice when an accurate diagnosis of more complex cases is necessary. To note, ES is known to bias coverages based on capture reagent and large rearrangements, which are extremely difficult to detect. Therefore, a recent publication reported that chromosomal microarray (CMA) testing followed by ES could improve the yield of genetic diagnosis [41, 42]. Analytical workflows for the diagnosis of GSD diseases are not fully standardized, so we recommended it, as shown in Fig. 3.

There were some limitations to the present study. First, the number of studies was very small, so the small sample size of patients restricted confidence in our analysis. Second, all included studies were observational and retrospectively selected cohort studies with the small number of patients, which could be resulted in the selection bias of patients. Finally, we only included English studies that result in missing the studies with other languages and not indexed in the databases.

## Conclusions

The correct characterization of clinical, biochemical, and pathological patterns of patients is important in order to interpret the genetic results. However, the MPS method could be a step forward in terms of enabling correct diagnosis of hepatic GSDs. All the patients mentioned in the analyzed reports, were offered genetic and metabolic assessments, including liver biopsy, enzyme assay, and single-gene sequencing prior to MPS. The present work demonstrates that, despite its cost, the time effectiveness and accuracy of MPS in the diagnosis of hepatic GSDs could avoid incorrect and/or delayed treatment of patients. We propose that TGS may be considered as the first-line method of choice for diagnosis of hepatic GSDs with a wide range of panel, as it allows the detection of pathogenic variants in GSD-associated vs. non-GSD-associated genes with overlapping symptoms in hepatic manifestations. It must be emphasized that with the extended use of TGS/ES strategies in finding the causes of liver disease, the so-called milder or adult forms of inborn errors can be accurately detected.

## Supplementary information

**Additional file1: Supplementary Table 1**. PRISMA 2009 Checklist. **Supplementary Table 2**. Search strategy for MEDLINE/PubMed, EMBASE, Cochrane Library, Scopus and Web of Science Core Collection databases. **Supplementary Table 3**. Quality assessment scores according to the NHLBI Quality Assessment Tool for Observational Cohort, Cross-Sectional and Case series Studies for each reviewer.

## Data Availability

The datasets used and/or analyzed during the current study are available from the corresponding author on reasonable request.

## Abbreviations

GSD: Glycogen storage diseases
MPS: Massively parallel sequencing
ES: Exome sequencing
TGS: Targeted gene sequencing
CMA: Chromosomal microarray
