# Supplementary material for: Diagnosis of hepatic glycogen storage disease patients with overlapping clinical symptoms by massively parallel sequencing: a systematic review of literature
Source: Orphanet J Rare Dis. 2020 Oct 14;15:286. doi: 10.1186/s13023-020-01573-8 (PMC7557034; doi:10.1186/s13023-020-01573-8)
Supplement: Supplementary file 1 — Additional file1: Supplementary Table 1. PRISMA 2009 Checklist. Supplementary Table 2. Search strategy for MEDLINE/PubMed, EMBASE, Cochrane Library, Scopus and Web of Science Core Collection databases. Supplementary Table 3. Quality assessment scores according to the NHLBI Quality Assessment Tool for Observational Cohort, Cross-Sectional and Case series Studies for each reviewer. [file 13023_2020_1573_MOESM1_ESM.pdf]

Diagnosis of hepatic Glycogen storage disease patients with overlapping clinical symptoms by massively parallel sequencing: a systematic review of literature

Zahra Beyzaei PhD [1], Bita Geramizadeh MD [1,2*], Sara Karimzadeh MSc[3]

[1]Transplant Research Center, Shiraz University of Medical Sciences, Shiraz, Iran.

[2]Department of Pathology, Shiraz University of Medical Sciences, Shiraz, Iran.

[3]Shiraz Medical School Library, Shiraz University of Medical Sciences, Shiraz, Iran.

*Corresponding Author:

Bita Geramizadeh

Professor of Pathology,

Department of Pathology

Shiraz University of Medical Sciences, Shiraz, Iran

Postal Address: Khalili St., Research Tower, Seventh Floor, Research Shiraz Transplant Research Center (STRC), Shiraz, Iran

Call number: 98-71-36472915

E-mail address: geramib@gmail.com

Supplementary Table 1. PRISMA 2009 Checklist

Section/topic	#	Checklist item	Reported on page #
**TITLE**			
Title	1	Identify the report as a systematic review, meta-analysis, or both.	1
**ABSTRACT**			
Structured summary	2	Provide a structured summary including, as applicable: background; objectives; data sources; study eligibility criteria, participants, and interventions; study appraisal and synthesis methods; results; limitations; conclusions and implications of key findings; systematic review registration number.	2
**INTRODUCTION**			
Rationale	3	Describe the rationale for the review in the context of what is already known.	3-4
Objectives	4	Provide an explicit statement of questions being addressed with reference to participants, interventions, comparisons, outcomes, and study design (PICOS).	4
**METHODS**			
Protocol and registration	5	Indicate if a review protocol exists, if and where it can be accessed (e.g., Web address), and, if available, provide registration information including registration number.	5
Eligibility criteria	6	Specify study characteristics (e.g., PICOS, length of follow-up) and report characteristics (e.g., years considered, language, publication status) used as criteria for eligibility, giving rationale.	5-6
Information sources	7	Describe all information sources (e.g., databases with dates of coverage, contact with study authors to identify additional studies) in the search and date last searched.	5
Search	8	Present full electronic search strategy for at least one database, including any limits used, such that it could be repeated.	Additional file 2
Study selection	9	State the process for selecting studies (i.e., screening, eligibility, included in systematic review, and, if applicable, included in the meta-analysis).	5,6
Data collection process	10	Describe method of data extraction from reports (e.g., piloted forms, independently, in duplicate) and any processes for obtaining and confirming data from investigators.	6
Data items	11	List and define all variables for which data were sought (e.g., PICOS, funding sources) and any assumptions and simplifications made.	7

Risk of bias in individual studies	12	Describe methods used for assessing risk of bias of individual studies (including specification of whether this was done at the study or outcome level), and how this information is to be used in any data synthesis.	6
Summary measures	13	State the principal summary measures (e.g., risk ratio, difference in means).	7
Synthesis of results	14	Describe the methods of handling data and combining results of studies, if done, including measures of consistency (e.g., I^2) for each meta-analysis.	None

Section/topic	#	Checklist item	Reported on page #
Risk of bias across studies	15	Specify any assessment of risk of bias that may affect the cumulative evidence (e.g., publication bias, selective reporting within studies).	6
Additional analyses	16	Describe methods of additional analyses (e.g., sensitivity or subgroup analyses, meta-regression), if done, indicating which were pre-specified.	None
**RESULTS**			
Study selection	17	Give numbers of studies screened, assessed for eligibility, and included in the review, with reasons for exclusions at each stage, ideally with a flow diagram.	7, Figure 1
Study characteristics	18	For each study, present characteristics for which data were extracted (e.g., study size, PICOS, follow-up period) and provide the citations.	7-9
Risk of bias within studies	19	Present data on risk of bias of each study and, if available, any outcome level assessment (see item 12).	7,Additional file 3
Results of individual studies	20	For all outcomes considered (benefits or harms), present, for each study: (a) simple summary data for each intervention group (b) effect estimates and confidence intervals, ideally with a forest plot.	7-9
Synthesis of results	21	Present results of each meta-analysis done, including confidence intervals and measures of consistency.	7-9
Risk of bias across studies	22	Present results of any assessment of risk of bias across studies (see Item 15).	None
Additional analysis	23	Give results of additional analyses, if done (e.g., sensitivity or subgroup analyses, meta-regression [see Item 16]).	None
**DISCUSSION**			
Summary of evidence	24	Summarize the main findings including the strength of evidence for each main outcome; consider their relevance to key groups (e.g., healthcare providers, users, and policy makers).	9, 11

Limitations	25	Discuss limitations at study and outcome level (e.g., risk of bias), and at review-level (e.g., incomplete retrieval of identified research, reporting bias).	11
Conclusions	26	Provide a general interpretation of the results in the context of other evidence, and implications for future research.	12
**FUNDING**			
Funding	27	Describe sources of funding for the systematic review and other support (e.g., supply of data); role of funders for the systematic review.	12

Supplementary Table 2. Search strategy for MEDLINE/PubMed, EMBASE, Cochrane Library, Scopus and Web of Science Core Collection databases

Pubmed	Search ((((((((((((((Next generation sequencing) OR whole exome sequencing) OR whole genome sequencing) OR targeted gene sequencing) OR Clinical exome sequencing) OR massively parallel sequencing) OR High Throughput Nucleotide Sequencing) OR Deep sequencing) OR High-Throughput DNA Sequencing)) OR "High-Throughput Nucleotide Sequencing"[Mesh]) OR "Whole Genome Sequencing"[Mesh]) OR "targeted gene sequencing "[Mesh]) OR "Whole Exome Sequencing"[Mesh])) AND (((((((((((((((((((((((((((Hepatorenal Glycogen Storage Disease) OR Glucosephosphatase deficiencies) OR glycogen storage disease type I) OR Von Gierke disease) OR glucose-6-phosphatase) OR glucose-6-phosphatase deficiency) OR Glucose-6-phosphate transporter) OR “G6PC”) OR “SLC37A4”) OR glycogen storage disease type III) OR “Cori disease”) OR glycogen debranching enzyme) OR debrancher deficiency) OR “AGL gene”) OR glycogen storage disease type IV) OR “Andersen disease”) OR “GBE1”) OR glycogen branching enzyme) OR Amylopectinosis) OR brancher deficiency) OR glycogen storage disease type VI) OR “liver glycogenphosphorylase”) OR Hepatic Glycogen Phosphorylase Deficiency) OR “PYGL”) OR “Hers' disease”) OR “hepatic glycogen storage disease”) OR “liver biopsy” OR “Glycogen Storage Disease Type IX”))) OR ((((((“Glycogen Storage Disease Type I"[Mesh]) OR "Glucose-6-Phosphatase"[Mesh])) OR "Glycogen Storage Disease Type III"[Mesh]) OR "Glycogen Debranching Enzyme System"[Mesh]) OR "Glycogen Storage Disease Type IV"[Mesh]) OR "Glycogen Storage Disease Type VI"[Mesh]))	104
Key 1	Search ((((((((((((Next generation sequencing) OR whole exome sequencing) OR whole genome sequencing) OR targeted gene sequencing) OR Clinical exome sequencing) OR massively parallel	103355

	sequencing) OR High Throughput Nucleotide Sequencing) OR Deep sequencing) OR High-Throughput DNA Sequencing)) OR "High-Throughput Nucleotide Sequencing"[Mesh]) OR "Whole Genome Sequencing"[Mesh]) OR "targeted gene sequencing "[Mesh]) OR "Whole Exome Sequencing"[Mesh])	
Key 2	Search ((((((((((((((((((((((((((Hepatorenal Glycogen Storage Disease) OR Glucosephosphatase deficiencies) OR glycogen storage disease type I) OR Von Gierke disease) OR glucose-6-phosphatase) OR glucose-6-phosphatase deficiency) OR Glucose-6-phosphate transporter) OR “G6PC”) OR “SLC37A4”) OR glycogen storage disease type III) OR “Cori disease”) OR glycogen debranching enzyme) OR debrancher deficiency) OR “AGL gene”) OR glycogen storage disease type IV) OR “Andersen disease”) OR “GBE1”) OR glycogen branching enzyme) OR Amylopectinosis) OR brancher deficiency) OR glycogen storage disease type VI) OR “liver glycogenphosphorylase”) OR Hepatic Glycogen Phosphorylase Deficiency) OR “PYGL”) OR “Hers' disease”) OR “hepatic glycogen storage disease”) OR “liver biopsy” OR “Glycogen Storage Disease Type IX”)) OR ((((((("Glycogen Storage Disease Type I"[Mesh]) OR "Glucose-6-Phosphatase"[Mesh])) OR "Glycogen Storage Disease Type III"[Mesh]) OR "Glycogen Debranching Enzyme System"[Mesh]) OR "Glycogen Storage Disease Type IV"[Mesh]) OR "Glycogen Storage Disease Type VI"[Mesh])	28517

Scopus	((TITLE-ABS-KEY (*next* AND *generation* AND *sequencing*) OR TITLE-ABS-KEY (*whole* AND *exome* AND *sequencing*) OR TITLE-ABS-KEY (*whole* AND *genome* AND *sequencing*) OR TITLE-ABS-KEY (*targeted* AND *gene* AND *sequencing*) OR TITLE-ABS-KEY (*clinical* AND *exome* AND *sequencing*) OR TITLE-ABS-	370

	KEY (*massively* AND *parallel* AND *sequencing*) OR TITLE-ABS-KEY (*high* AND *throughput* AND *nucleotide* AND *sequencing*) OR TITLE-ABS-KEY (*deep* AND *sequencing*) OR TITLE-ABS-KEY (*high-throughput* AND *dna* AND *sequencing*))) AND ((TITLE-ABS-KEY (*hepatorenal* AND *glycogen* AND *storage* AND *disease*) OR TITLE-ABS-KEY (*glucosephosphatase* AND *deficiencies*) OR TITLE-ABS-KEY (*glycogen* AND *storage* AND *disease* AND *type* AND *i*) OR TITLE-ABS-KEY (*von* AND *gierke* AND *disease*) OR TITLE-ABS-KEY (*glucose-6-phosphatase*) OR TITLE-ABS-KEY (*glucose-6-phosphatase* AND *deficiency*) OR TITLE-ABS-KEY (*glucose-6-phosphate* AND *transporter*) OR TITLE-ABS-KEY (*"G6PC"*) OR TITLE-ABS-KEY (*"SLC37A4"*) OR TITLE-ABS-KEY (*glycogen* AND *storage* AND *disease* AND *type* AND *iii*) OR TITLE-ABS-KEY (*"Cori disease"*) OR TITLE-ABS-KEY (*glycogen* AND *debranching* AND *enzyme*) OR TITLE-ABS-KEY (*debrancher* AND *deficiency*) OR TITLE-ABS-KEY (*"AGL gene"*) OR TITLE-ABS-KEY (*glycogen* AND *storage* AND *disease* AND *type* AND *iv*) OR TITLE-ABS-KEY (*"Andersen disease"*) OR TITLE-ABS-KEY (*"GBE1"*) OR TITLE-ABS-KEY (*glycogen* AND *branching* AND *enzyme*) OR TITLE-ABS-KEY (*amylopectinosis*) OR TITLE-ABS-KEY (*brancher* AND *deficiency*) OR TITLE-ABS-KEY (*glycogen* AND *storage* AND *disease* AND *type* AND *vi*) OR TITLE-ABS-KEY (*"liver glycogenphosphorylase"*) OR TITLE-ABS-KEY (*hepatic* AND *glycogen* AND *phosphorylase* AND *deficiency*) OR TITLE-ABS-KEY (*"PYGL"*) OR TITLE-ABS-KEY (*"Hers' disease"*) OR TITLE-ABS-KEY (*"hepatic glycogen storage disease"*) OR TITLE-ABS-KEY (*"liver biopsy"*) **OR TITLE-ABS-KEY** (“Glycogen Storage Disease Type IX”**)**))	

Key 1	(TITLE-ABS-KEY (*next* AND *generation* AND *sequencing*) OR TITLE-ABS-KEY (*whole* AND *exome* AND *sequencing*) OR TITLE-ABS-KEY (*whole* AND *genome* AND *sequencing*) OR TITLE-ABS-KEY (*targeted* AND *gene* AND *sequencing*) OR TITLE-ABS-KEY (*clinical* AND *exome* AND *sequencing*) OR TITLE-ABS-KEY (*massively* AND *parallel* AND *sequencing*) OR TITLE-ABS-KEY (*high* AND *throughput* AND *nucleotide* AND *sequencing*) OR TITLE-ABS-KEY (*deep* AND *sequencing*) OR TITLE-ABS-KEY (*high-throughput* AND *dna* AND *sequencing*))	125042
Key 2	(TITLE-ABS-KEY (*hepatorenal* AND *glycogen* AND *storage* AND *disease*) OR TITLE-ABS-KEY (*glucosephosphatase* AND *deficiencies*) OR TITLE-ABS-KEY (*glycogen* AND *storage* AND *disease* AND *type* AND *i*) OR TITLE-ABS-KEY (*von* AND *gierke* AND *disease*) OR TITLE-ABS-KEY (*glucose-6-phosphatase*) OR TITLE-ABS-KEY (*glucose-6-phosphatase* AND *deficiency*) OR TITLE-ABS-KEY (*glucose-6-phosphate* AND *transporter*) OR TITLE-ABS-KEY (*"G6PC"*) OR TITLE-ABS-KEY (*"SLC37A4"*) OR TITLE-ABS-KEY (*glycogen* AND *storage* AND *disease* AND *type* AND *iii*) OR TITLE-ABS-KEY (*"Cori disease"*) OR TITLE-ABS-KEY (*glycogen* AND *debranching* AND *enzyme*) OR TITLE-ABS-KEY (*debrancher* AND *deficiency*) OR TITLE-ABS-KEY (*"AGL gene"*) OR TITLE-ABS-KEY (*glycogen* AND *storage* AND *disease* AND *type* AND *iv*) OR TITLE-ABS-KEY (*"Andersen disease"*) OR TITLE-ABS-KEY (*"GBE1"*) OR TITLE-ABS-KEY (*glycogen* AND *branching* AND *enzyme*) OR TITLE-ABS-KEY (*amylopectinosis*) OR TITLE-ABS-KEY (*brancher* AND *deficiency*) OR TITLE-ABS-KEY (*glycogen* AND *storage* AND *disease* AND *type* AND *vi*) OR TITLE-ABS-KEY (*"liver*	65977

	glycogenphosphorylase") OR TITLE-ABS-KEY (*hepatic* AND *glycogen* AND *phosphorylase* AND *deficiency*) OR TITLE-ABS-KEY (*"PYGL"*) OR TITLE-ABS-KEY (*"Hers' disease"*) OR TITLE-ABS-KEY (*"hepatic glycogen storage disease"*) OR TITLE-ABS-KEY (*"liver biopsy"*) OR TITLE-ABS-KEY (“Glycogen Storage Disease Type IX”)	

Embase	(next AND generation AND sequencing OR (whole AND exome AND sequencing) OR (whole AND genome AND sequencing) OR (targeted AND gene AND sequencing) OR (clinical AND exome AND sequencing) OR (massively AND parallel AND sequencing) OR (high AND throughput AND nucleotide AND sequencing) OR (deep AND sequencing) OR ('high throughput' AND dna AND sequencing)) AND (hepatorenal AND glycogen AND storage AND disease OR (glucosephosphatase AND deficiencies) OR (glycogen AND storage AND disease AND type AND i) OR (von AND gierke AND disease) OR 'glucose 6 phosphatase' OR (glycogen AND storage AND disease AND type AND iii) OR (cori AND disease) OR (glycogen AND debranching AND enzyme) OR (debrancher AND deficiency) OR (glycogen AND storage AND disease AND type AND iv) OR (andersen AND disease) OR amylopectinosis OR (brancher AND deficiency) OR (glycogen AND storage AND disease AND type AND vi) OR (Glycogen AND Storage AND Disease AND Type AND IX))	941
Key1	'next generation sequencing technology' OR 'whole exome sequencing' OR (whole AND genome AND sequencing) OR (targeted AND gene AND sequencing) OR 'clinical exome sequencing' OR (massively AND parallel AND sequencing) OR (high AND throughput AND nucleotide AND sequencing) OR (deep AND sequencing) OR ('high throughput' AND dna AND sequencing)	112214

Key2	hepatorenal AND glycogen AND storage AND disease OR (glucosephosphatase AND deficiencies) OR (glycogen AND storage AND disease AND type AND i) OR (von AND gierke AND disease) OR 'glucose 6 phosphatase' OR ('glucose 6 phosphatase' AND deficiency) OR ('glucose 6 phosphate' AND transporter) OR 'g6pc' OR 'slc37a4' OR (glycogen AND storage AND disease AND type AND iii) OR (cori AND disease) OR (glycogen AND debranching AND enzyme) OR (debrancher AND deficiency) OR 'agl gene' OR (glycogen AND storage AND disease AND type AND iv) OR (andersen AND disease) OR (glycogen AND branching AND enzyme) OR amylopectinosis OR (brancher AND deficiency) OR (glycogen AND storage AND disease AND type AND vi OR (liver AND glycogenphosphorylase) OR (hepatic AND glycogen AND phosphorylase AND deficiency) OR (hers AND disease) OR (hepatic AND glycogen AND storage AND disease) OR (liver AND biopsy) OR (Glycogen Storage Disease Type IX))	143294

ISI	**(TOPIC:** (Next generation sequencing) *OR* **TOPIC:** (whole exome sequencing) *OR* **TOPIC:** (whole genome sequencing) *OR* **TOPIC:** (targeted gene sequencing) *OR* **TOPIC:** (Clinical exome sequencing) *OR* **TOPIC:** (massively parallel sequencing) *OR* **TOPIC:** (High Throughput Nucleotide Sequencing) *OR* **TOPIC:** (Deep sequencing) *OR* **TOPIC:** (High-Throughput DNA Sequencing)<br><br>Indexes=SCI-EXPANDED, SSCI, CPCI-S, CPCI-SSH, ESCI Timespan=All years) AND	271

	**(TOPIC:** (Hepatorenal Glycogen Storage Disease) *OR* **TOPIC:** (Glucosephosphatase deficiencies) *OR* **TOPIC:** (glycogen storage disease type I) *OR* **TOPIC:** (Von Gierke disease) *OR* **TOPIC:** (glucose-6-phosphatase) *OR* **TOPIC:** (glucose-6-phosphatase deficiency) *OR* **TOPIC:** (Glucose-6-phosphate transporter) *OR* **TOPIC:** ("G6PC") *OR* **TOPIC:** ("SLC37A4") *OR* **TOPIC:** (glycogen storage disease type III) *OR* **TOPIC:** (Cori disease) *OR* **TOPIC:** (glycogen debranching enzyme) *OR* **TOPIC:** (debrancher deficiency) *OR* **TOPIC:** ("AGL gene") *OR* **TOPIC:** (glycogen storage disease type IV) *OR* **TOPIC:** (Andersen disease) *OR* **TOPIC:** (glycogen branching enzyme) *OR* **TOPIC:** (Amylopectinosis) *OR* **TOPIC:** (brancher deficiency) *OR* **TOPIC:** (glycogen storage disease type VI) *OR* **TOPIC:** (liver glycogenphosphorylase) *OR* **TOPIC:** (Hepatic Glycogen Phosphorylase Deficiency) *OR* **TOPIC:** (Hers' disease) *OR* **TOPIC:** (hepatic glycogen storage disease) *OR* **TOPIC:** (liver biopsy) *OR* **TOPIC:** (**Glycogen Storage Disease Type IX)**<br><br>Indexes=SCI-EXPANDED, SSCI, CPCI-S, CPCI-SSH, ESCI Timespan=All years)	
Key 1	**(TOPIC:** (Next generation sequencing) *OR* **TOPIC:** (whole exome sequencing) *OR* **TOPIC:** (whole genome sequencing) *OR* **TOPIC:** (targeted gene sequencing) *OR* **TOPIC:** (Clinical exome sequencing) *OR* **TOPIC:** (massively parallel sequencing) *OR* **TOPIC:** (High Throughput Nucleotide Sequencing) *OR* **TOPIC:** (Deep sequencing) *OR* **TOPIC:** (High-Throughput DNA Sequencing)<br><br>Indexes=SCI-EXPANDED, SSCI, CPCI-S, CPCI-SSH, ESCI Timespan=All years)	208264
Key 2	**(TOPIC:** (Hepatorenal Glycogen Storage Disease) *OR* **TOPIC:** (Glucosephosphatase deficiencies) *OR* **TOPIC:** (glycogen storage disease type I) *OR* **TOPIC:** (Von Gierke disease) *OR* **TOPIC:** (glucose-6-phosphatase) *OR* **TOPIC:** (glucose-6-phosphatase deficiency) *OR* **TOPIC:** (Glucose-6-phosphate	43539

	transporter) *OR* **TOPIC:** ("G6PC") *OR* **TOPIC:** ("SLC37A4") *OR* **TOPIC:** (glycogen storage disease type III) *OR* **TOPIC:** (Cori disease) *OR* **TOPIC:** (glycogen debranching enzyme) *OR* **TOPIC:** (debrancher deficiency) *OR* **TOPIC:** ("AGL gene") *OR* **TOPIC:** (glycogen storage disease type IV) *OR* **TOPIC:** (Andersen disease) *OR* **TOPIC:** (glycogen branching enzyme) *OR* **TOPIC:** (Amylopectinosis) *OR* **TOPIC:** (brancher deficiency) *OR* **TOPIC:** (glycogen storage disease type VI) *OR* **TOPIC:** (liver glycogenphosphorylase) *OR* **TOPIC:** (Hepatic Glycogen Phosphorylase Deficiency) *OR* **TOPIC:** (Hers' disease) *OR* **TOPIC:** (hepatic glycogen storage disease) *OR* **TOPIC:** (liver biopsy) *OR* **TOPIC:** (Glycogen Storage Disease Type IX)<br><br>Indexes=SCI-EXPANDED, SSCI, CPCI-S, CPCI-SSH, ESCI Timespan=All years)	

Cochrane	"next generation sequencing" in Title Abstract Keyword OR whole exome sequencing in Title Abstract Keyword OR whole genome sequencing in Title Abstract Keyword OR targeted gene sequencing in Title Abstract Keyword OR Clinical exome sequencing in Title Abstract Keyword - (Word variations have been searched)'	6
	"next generation sequencing" in Title Abstract Keyword AND Hepatorenal Glycogen Storage Disease in Title Abstract Keyword	0
	"next generation sequencing" in Title Abstract Keyword AND Von Gierke disease in Title Abstract Keyword	0

Supplementary Table 3. Quality assessment scores according to the NHLBI Quality Assessment Tool for Observational Cohort, Cross-Sectional and Case series Studies for each reviewer.

Reviewer 1:

Study	Q1	Q2	Q3	Q4	Q5	Q6	Q7	Q8	Q9	Q10	Q11	Q12	Q13	Q14	Quality Rating
**Yang et al. 2019**	YES	YES	NO	YES	NO	NO	NA	NA	YES	NA	YES	NR	YES	NR	POOR
**Tong et al. 2018**	YES	YES	YES	YES	NO	YES	NA	NA	NR	NA	YES	YES	YES	YES	GOOD
**Zhang et al. 2017**	YES	YES	YES	YES	NO	NO	NA	NA	YES	NA	YES	YES	YES	YES	GOOD
**Skakik et al. 2017**	YES	YES	YES	YES	NO	YES	NA	NA	YES	NA	YES	NR	YES	YES	GOOD
**Choi et al. 2017**	YES	YES	NO	YES	NO	NO	NA	NA	YES	NA	YES	NR	YES	NR	POOR
**Vega et al. 2016**	YES	YES	YES	YES	NO	YES	NA	NA	YES	NA	YES	NO	YES	YES	GOOD
**Wang et al. 2016**	YES	YES	YES	YES	NO	NO	NA	NA	YES	NA	YES	NO	YES	NR	FAIR
**Rousseau-Nepton et al. 2015**	YES	YES	NO	YES	NO	NO	NA	NA	YES	NA	YES	NR	YES	NR	POOR
**Fahiminiya 2014**	YES	YES	NO	YES	NO	NO	NA	NA	YES	NA	YES	NR	YES	YES	FAIR
**Roscher et al. 2014**	YES	YES	YES	YES	NO	YES	NA	NA	NR	NA	YES	NR	YES	NR	FAIR
**Wang et al. 2013**	YES	YES	YES	YES	NO	NO	NA	NA	YES	NA	YES	NO	YES	YES	FAIR

NHLBI National Heart, Lung, and Blood Institute; *CD* cannot be determined; *NA* not applicable; *NR* not reported;

“Continue Table 3”

Reviewer 2:

Study	Q1	Q2	Q3	Q4	Q5	Q6	Q7	Q8	Q9	Q10	Q11	Q12	Q13	Q14	Quality Rating
**Yang et al. 2019**	YES	YES	YES	YES	NO	NO	NA	NA	YES	NA	YES	NR	YES	NR	FAIR
**Tong et al. 2018**	YES	YES	YES	YES	NO	YES	NA	NA	NR	NA	YES	YES	YES	YES	GOOD
**Zhang et al. 2017**	YES	YES	YES	YES	NO	NO	NA	NA	YES	NA	YES	YES	YES	YES	GOOD
**Skakik et al. 2017**	YES	YES	YES	YES	NO	YES	NA	NA	YES	NA	YES	NR	YES	YES	GOOD
**Choi et al. 2017**	YES	YES	NO	YES	NO	NO	NA	NA	YES	NA	YES	NR	YES	NR	POOR
**Vega et al. 2016**	YES	YES	YES	YES	NO	YES	NA	NA	YES	NA	YES	NO	YES	YES	GOOD
**Wang et al. 2016**	YES	YES	YES	YES	NO	NO	NA	NA	YES	NA	YES	NO	YES	NR	FAIR
**Rousseau-Nepton et al. 2015**	YES	YES	NO	YES	NO	NO	NA	NA	YES	NA	YES	NR	YES	NR	POOR
**Fahiminiya 2014**	YES	YES	YES	YES	NO	NO	NA	NA	YES	NA	YES	NR	YES	YES	GOOD
**Roscher et al. 2014**	YES	YES	YES	YES	NO	YES	NA	NA	NR	NA	YES	NR	YES	NR	FAIR
**Wang et al. 2013**	YES	YES	YES	YES	NO	NO	NA	NA	YES	NA	YES	NO	YES	YES	FAIR

NHLBI National Heart, Lung, and Blood Institute; *CD* cannot be determined; *NA* not applicable; *NR* not reported;
